# Supplementary material for: Rapid Bayesian optimisation for synthesis of short polymer fiber materials
Source: Sci Rep. 2017 Jul 18;7:5683. doi: 10.1038/s41598-017-05723-0 (PMC5515927; doi:10.1038/s41598-017-05723-0)
Supplement: Supplementary file 1 — Supplementary Materials [file 41598_2017_5723_MOESM1_ESM.pdf]

Rapid Bayesian optimisation for synthesis of short polymer fiber materials

Cheng Li[1], David Rubin De Celis Leal[2], Santu Rana[1], Sunil Gupta[1], Alessandra Sutti[2], Stewart Greenhill[1*], Teo Slezak[2], Murray Height[3], and Svetha Venkatesh[1]

[1]Centre for Pattern Recognition and Data Analytics (PRaDA), Deakin University, Victoria, Australia
[2]Institute for Frontier Materials (IFM), Deakin University, Victoria, Australia
[3]HeiQ Australia, Pty Ltd
[*]s.greenhill@deakin.edu.au

Supplementary Materials

Algorithm parameters

We use the SE kernel as the kernel function of a Gaussian process and Expected Improvement as the acquisition function[1]. Two hyperparameters $\Theta = \{\theta_q, \theta\}$ are automatically estimated at each iteration, where θ_q is the length-scale of the Gaussian kernel and the variance of Gaussian noise for learning the latent quality scores; and θ is the length-scale of SE kernel in Bayesian optimization. The θ_q can be estimated by maximizing the evidence approximation[2]. The θ can be estimated by maximizing the marginal likelihood under Gaussian process[1]. In this experiment, we empirically set the noise variance as 0.1 and the signal variance as 0.25.

Correlations between input parameters and fiber properties

Table S.1 shows correlations between experimental settings and product parameters. Correlation coefficients R result from performing a second order polynomical fit using samples from all 9 experiments. The combined R is the product of the correlations for length and diameter.

Sensitivity analysis was done following the methods in[3–5]. Table 1 presents average first-order effects of each parameter for combined quantity across Run #1-5 (Target 1). 3 of 5 Runs are highly correlated with the average effects (correlation>0.8 for Run #1, 3, 5). Considering the average effects, the most significant parameter is solvent speed, the least significant parameter is angle and the others have moderate effects.

Further experimental results

Figure S.2 shows the results obtained using a target fiber of length 50μm and diameter 0.4μm. With these criteria it can be seen that it is not possible to achieve both length and diameter targets contemporaneously. The sample at iteration 2 satisfies the length target (within 2.7%, a value which sits comfortably within experimental and measurement error), but a better overall solution cannot be found, leaving a large difference to the diameter target (104%). Note that sample 12 offered an improved diameter (82%) but at the expense of a poorer overall quality.

Parameter	Length		Diameter		Combined	
	R	rank	R	rank	R	rank
Solvent Speed	0.58	**1**	0.46	**2**	0.27	**1**
Polymer Flow	0.37	**2**	0.03	(4)	0.01	(3)
Channel Width	0.34	**3**	0.51	**1**	0.17	**2**
Angle	0.06	(5)	0.01	(5)	0.00	(5)
Position	0.10	(4)	0.06	(3)	0.01	(4)

Table S.1. Correlation coefficient (R) of each input parameter with Length and Diameter of produced fibers, and the rank of each parameter in order from most to least significant.

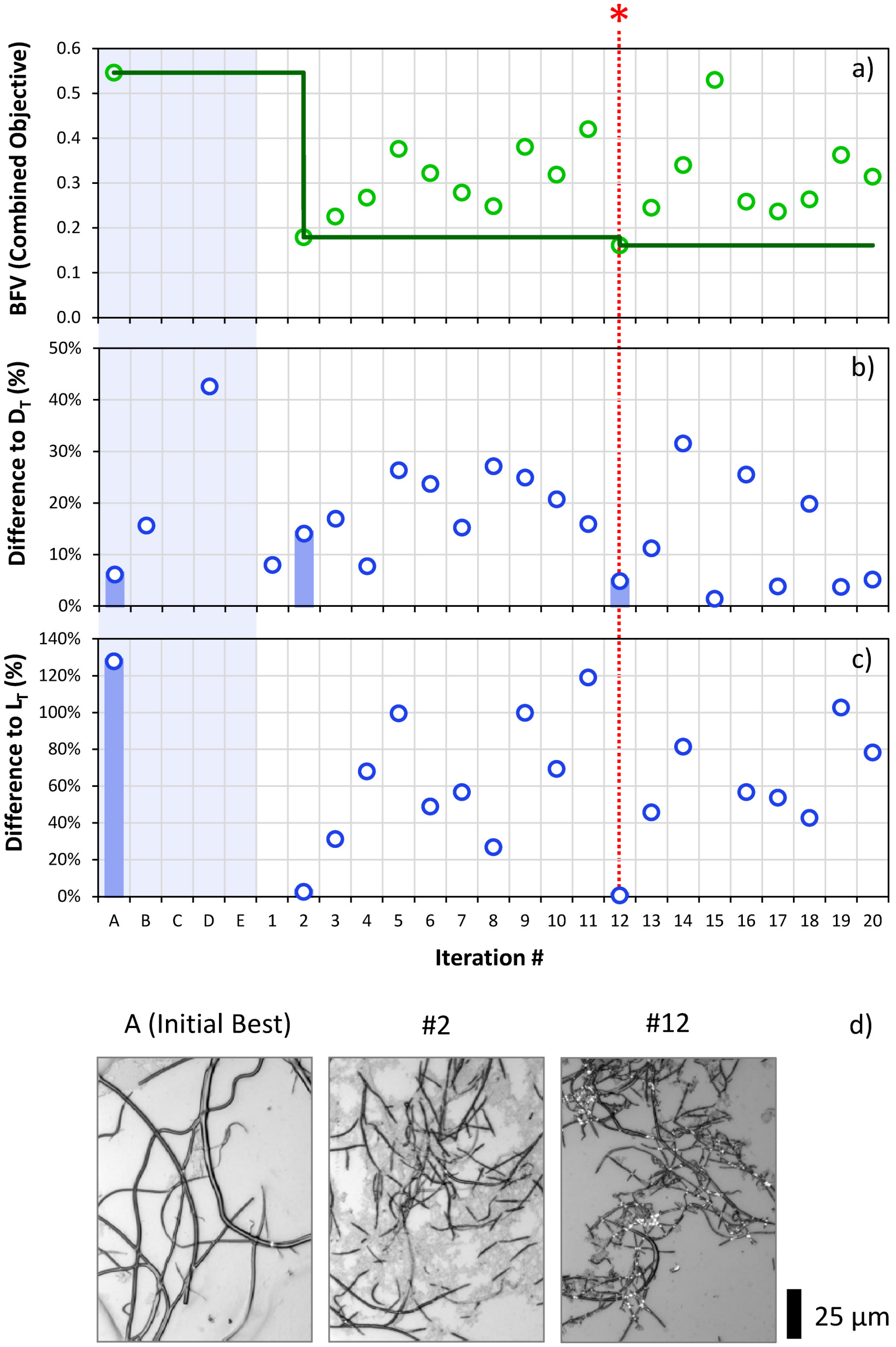

Figure S.1. The results of Run 4 starting from all bad samples for Target 1 (L=70µm, D=1.0µm) showing (a) the Best Found Value, (b) deviation in length *L* from target (c) deviation in diameter *D* from target (d) The corresponding fiber images.

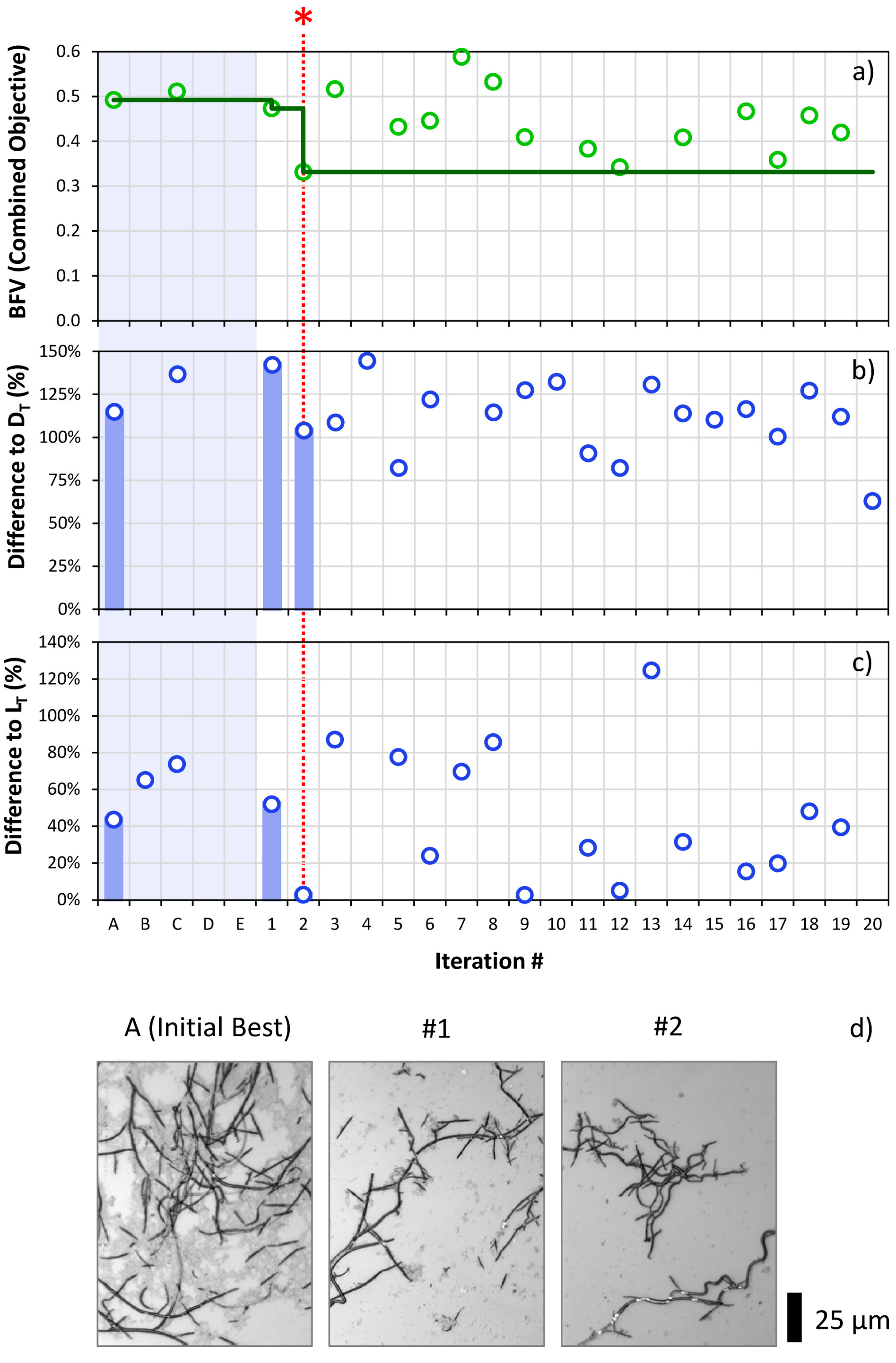

Figure S.2. The results of Run 9 starting from random samples for Target 3 (L=50µm, D=0.4µm) showing (a) the BFV, (b) deviation in length *L* from target (c) deviation in diameter *D* from target (d) The corresponding SPF optical microscope images.

Parameter	Average effects (%)
Solvent Speed	33.52
Polymer Flow	13.04
Channel Width	12.24
Angle	1.94
Position	16.95

Table S.2. The average first-order effects of parameters for combined quantity

.

References

1. Snoek, J., Larochelle, H. & Adams, R. P. Practical Bayesian optimization of machine learning algorithms. In *Advances in Neural Information Processing Systems NIPS*, 2960–2968 (2012).
2. Chu, W. & Ghahramani, Z. Preference learning with Gaussian processes. In *Proceedings of the 22nd International Conference on Machine Learning*, ICML '05, 137–144 (ACM, New York, NY, USA, 2005).
3. O'Hagan, A. Bayesian analysis of computer code outputs: A tutorial. *Reliab. Eng. & Syst. Saf.* **91**, 1290 – 1300 (2006). URL http://www.sciencedirect.com/science/article/pii/S0951832005002383. DOI https://doi.org/10.1016/j.ress.2005.11.025.
4. Oakley, J. E. & O'Hagan, A. Probabilistic sensitivity analysis of complex models: A Bayesian approach. *J. Royal Stat. Soc. Ser. B (Statistical Methodol.* **66**, 751–769 (2004).
5. Vu-Bac, N., Lahmer, T., Zhuang, X., Nguyen-Thoi, T. & Rabczuk, T. A software framework for probabilistic sensitivity analysis for computationally expensive models. *Adv. Eng. Softw.* **100**, 19 – 31 (2016).
